# Microbial quality of reduced‐sodium napa cabbage kimchi and its processing

**DOI:** 10.1002/fsn3.898

**Published:** 2019-01-24

**Authors:** Won‐Jae Song, Ha‐Yull Chung, Dong‐Hyun Kang, Jae‐Won Ha

**Affiliations:** ^1^ Department of Agricultural Biotechnology Center for Food Safety and Toxicology Center for Food and Bioconvergence, and Research Institute for Agricultural and Life Sciences Seoul National University Seoul Korea; ^2^ Department of Food Science and Biotechnology College of Engineering, Food & Bio‐industry Research Center Hankyong National University Anseong‐si Korea

**Keywords:** kimchi, low‐salt food, microbial safety, processing, reduced‐sodium

## Abstract

This study evaluated the microbial safety of reduced‐sodium napa cabbage kimchi products by comparing with conventional kimchi samples. Five commercial kimchi samples were collected from different manufacturers in Korea. Total aerobic plate counts and coliforms counts between regular and reduced‐sodium kimchi were not significantly (*p *>* *0.05) different and major foodborne pathogens, including *Escherichia coli* O157:H7, *Salmonella* spp.*, Listeria monocytogenes*,* Staphylococcus aureus*, and *Yersinia enterocolitica* were not detected in any sample. *Bacillus cereus* contamination among all kimchi samples was less than the regulation level (3.0 log CFU/g). However, high levels of coliforms were observed in both types of samples. To investigate microbial hazards of kimchi processing, we analyzed specific kimchi production processes and found five control points which can reduce coliform levels in kimchi samples. The results of this study could be helpful for the kimchi industry to produce safe reduced‐sodium kimchi products.

## 1 INTRODUCTION

Kimchi is one of the most popular traditional Korean fermented foods. In recent years, kimchi has attracted attention because consumers are increasingly interested in health and nutrition. Kimchi contains high levels of vitamins, minerals, dietary fiber, and phytochemicals including benzyl isothiocyanate, indole compounds, and thiocyanate (Park, Jeong, Lee, & Daily, 2014). Also, kimchi consumption has many reported health benefits like antimutagenic, anticancer, antiobesity, antioxidative, antiaging effects, and cholesterol‐lowering activities (Park et al., 2014). But, unfortunately, there have been several outbreaks traced to foodborne pathogen‐contaminated kimchi. An outbreak of *Escherichia coli* O99 and O120 reported in 2012 from Gyeonggi Province, Republic of Korea, was associated with consumption of contaminated kimchi which resulted in 1193 illnesses (Cho, Joo, et al., 2014). In 2012, 1642 cases of *E. coli* O169 infections linked to kimchi consumption were reported (Cho, Kim, et al., 2014). There was also an outbreak of *E. coli* O6 infections in South Korea in 2013 and 2014 linked to kimchi (Shin et al., 2016). Also, Choi, Lee, Kim, Lee, Kim, Lee, Ha, Oh, Choi, et al. (2018) reported that pathogenic *E. coli* and *Salmonella* could survive for over 12 days at 4°C in kimchi. HACCP is a food safety program to ensure safety from raw materials to final product. In 2006, the Korean government announced that kimchi processing should be managed by a HACCP program (Park & Ha, 2017). However, unfortunately, there have been several outbreaks due to foodborne pathogen‐contaminated kimchi as we mentioned above.

Sodium is the most abundant component in extracellular fluids and allows the transport of nutrients and is an essential ingredient which contributes to blood pressure regulation and transmission of nerve impulses (Cruz et al., 2011). But excess sodium intake results in high blood pressure which is a risk factor for cardiovascular disease (Lawes, Hoorn, & Rodgers, 2008). For this reason, many researchers have reported the beneficial health effect of a low‐sodium diet. Dall et al. (2009) reported that low‐sodium intake reduced the risk of hypertension, cerebrovascular disease, congestive heart failure, and coronary heart disease. Usually, traditional Korean foods based on kimchi contain a high amount of salt. Koreans typically ingest 13.4 g of salt per day which is much higher than the WHO recommendation of <5 g per day (Lee et al., 2011). To limit sodium consumption, reduced‐sodium traditional Korean foods are being developed. However, reducing the sodium content may result in increased water activity (a_w_) of foods. a_w_ is one of the key factors which greatly affect the microbial safety of foods, and low a_w_ can prevent the growth of microorganisms. Therefore, the microbial safety of reduced‐sodium foods needs to be investigated.

Indicator microorganisms are used as a marker of microbial quality in foods and food processing (Chapin, Nightingale, Worobo, Wiedmann, & Strawn, 2014). The presence of indicator microorganisms can reveal potential pathogen contamination of foods (Buchanan & Oni, 2012). Coliforms are one of the indicator microorganisms which can be used in assessment of product production (Zoellner et al., 2016). But, unfortunately, a study which confirmed that kimchi processing is not free from microbial contaminations has been reported. Kim and Yoon (2005) reported that coliforms contamination occurred during kimchi production. Although they used coliforms‐free raw materials for making kimchi, coliforms were detected during ripening. Analyzing the ongoing processing protocol to identify microbial hazards is required. And also control methods for reducing microbial hazards during kimchi processing are required.

For these reasons, our objective was to compare the microbial quality of regular and reduced‐sodium kimchi products and to evaluate the microbial hazards of kimchi processing and identify five control points for reducing coliforms during kimchi production.

## 2 MATERIALS AND METHODS

### 2.1 Preparation of samples

Commercial napa cabbage kimchi samples were collected from various companies and locations (Cheongyang‐gun, Daejeon, Changwon, Bucheon, and Paju) in Korea. The kimchi samples were designated as A, B, C, D, and E. All samples were collected within 2 days of manufacture. All samples were examined immediately after delivery.

### 2.2 Measurement of pH and a_w_

To measure pH and a_w_, 50 g of kimchi sample was ground with a blender. pH was measured with a pH meter (Mettler‐Toledo, Switzerland). Water activity of kimchi was measured with an Aqualab model 4TE a_w_ meter (METER Group, Inc., Pullman, WA, USA).

### 2.3 Total aerobic plate counts and coliforms

For enumeration of total aerobic plate counts (APC) and coliforms, 25 g of each kimchi sample was transferred to a sterile stomacher bag (Labplas Inc., Sainte‐Julie, Quebec, Canada) containing 225 ml of sterile 0.2% peptone water (PW; Difco) and homogenized for 2 min in a stomacher (EASY MIX, AES Chemunex, Rennes, France). After homogenization, 1 ml aliquots of homogenized samples were tenfold serially diluted in 9 ml of sterile 0.2% PW, and 1 ml of sample or diluent was plated onto 3M Petrifilm Aerobic Count Plates and 3M Petrifilm *E. coli*/Coliform Count Plates (3M Health Care, MN, USA). The plates were incubated at 32°C for 48 hr (Aerobic Count Plates) and 35°C (*E. coli*/Coliform Count Plates) for 24–48 hr, respectively.

### 2.4 Isolation of *E. coli* O157:H7

The method of McEvoy et al. (2003) was used for isolation of *E. coli* O157:H7. Twenty‐five gram of kimchi was transferred to a sterile stomacher bag containing 225 ml of modified *E. coli* broth with novobiocin (mEC Broth; Merck), homogenized for 2 min with a stomacher and enriched by incubating at 37°C for 24 hr. One loop of the enrichment broth was streaked onto a plate of Sorbitol MacConkey Agar with cefixime (0.05 mg/L) and potassium tellurite (2.5 mg/L) (CT‐SMAC, Oxoid) and incubated at 37°C for 24 hr. For a second isolation, colonies formed on the CT‐SMAC agar plate were streaked onto eosin methylene blue agar plates (EMB, Becton, Dickinson Co.) and phenol red agar with 4‐methylumbelliferyl‐β‐D‐glucuronide (PRS‐MUG) and incubated at 37°C for 24 hr. Presumptive positive isolates on EMB and PRS‐MUG agar were tested for O157 and H7 antigens using the latex agglutination test (Remel^™^ Wellcox^™^ *E. coli* O157:H7). Latex agglutination‐positive isolates were subjected to the following tests: H_2_S production, indole production, Voges–Proskauer test, citrate utilization and lysine decarboxylase and ornithine decarboxylase test by using API 20E biochemical test strips (bioMérieux, La Balme les Grottes, France), cellobiose fermentation by API 50CHL medium (bioMérieux, La Balme les Grottes, France), and carbohydrate fermentation in triple sugar iron agar slants (TSI, Becton, Dickinson Co.).

### 2.5 Isolation of *Salmonella* spp.

The isolation method for *Salmonella* spp., described by Lee, Runyon, Herrman, Phillips, and Hsiehm (2015), was followed, which includes two enrichment steps. First, 25 g of kimchi sample was transferred to a sterile stomacher bag (Labplas Inc., Sainte‐Julie, Quebec, Canada) containing 225 ml of buffered peptone water (Oxoid), homogenized for 2 min in a stomacher, and incubated at 37°C for 24 hr. Following incubation, 0.1 ml of the culture was transferred into 10 ml of Rappaport–Vassiliadis Broth (Difco) for the second enrichment and then incubated at 42°C for 24 hr. One loop of the second enrichment broth was spread onto a Xylose Lysine Desoxycholate Agar (XLD; Difco) and incubated at 37°C for 24 hr. Presumptive positive colonies on the XLD agar plates were transferred to a TSI agar slant (Becton, Dickinson Co.) and incubated at 37°C for 24 hr. Presumptive positive colonies further characterized on TSI agar slants were selected and subjected to glucose, H_2_S, urease, lysine decarboxylase, indole, and Voges–Proskauer tests by using API 20E biochemical test strips, the malonate test by using malonate broth (Difco^™^, Becton Dickson and Co., Sparks, MD, USA), and the potassium cyanide test by using KCN broth with KCN supplement (MB cell, LA, USA).

### 2.6 Isolation of *Listeria monocytogenes*

For the detection of *L. monocytogenes,* we followed the FDA BAM method described in Gasanov, Hughes, and Hansbro (2005). Twenty‐five gram of kimchi was transferred to a sterile stomacher bag containing 225 ml of *Listeria* enrichment broth (Difco), homogenized for 2 min in a stomacher, and incubated at 30°C for 48 hr. One loop of the enrichment broth was streaked onto an Oxford Agar Base plate with antimicrobial supplement (OAB; MB Cell) and incubated at 30°C for 24–48 hr. Presumptive positive colonies on OAB agar plates were selected for *L. monocytogenes* confirmation using the API Listeria (bioMérieux, La Balme les Grottes, France) test.

### 2.7 Isolation of *Staphylococcus aureus*

Twenty‐five gram of kimchi was transferred to a sterile stomacher bag containing 225 ml of 10% NaCl Tryptic Soy Broth (TSB; Difco, BD, Sparks, MD), homogenized for 2 min in a stomacher and incubated at 37°C for 24 hr. One loop of the enriched broth was streaked onto a Baird–Parker agar plate (BPA; Difco) and incubated at 37°C for 24 hr. Presumptive positive colonies formed on BPA agar plates were selected and subjected to Gram staining and the catalase test. Gram‐ and catalase‐positive isolates were identified using API Staph system (bioMérieux, La Balme les Grottes, France) (Normanno et al., 2007).

### 2.8 Isolation of *Yersinia enterocolitica*

Twenty‐five gram of kimchi sample was transferred to a sterile stomacher bag (Labplas Inc., Sainte‐Julie, Quebec, Canada) containing 225 ml of Peptone Sorbitol Bile Broth (PSBB; MB cell), homogenized for 2 min in a stomacher, and incubated at 25°C for 2 days. Following incubation, 0.1 ml of the culture was mixed into 1 ml of 0.5% NaCl solution containing 0.5% KOH. One loop of the mixture was spread onto a Cefsulodin–Irgasan–Novobiocin agar plate (CIN; MB cell) and incubated at 30°C for 24 hr. Presumptive positive colonies formed on CIN agar plates were transferred to Kligler Agar and Christensen's Urea Agar (Merck, Darmstadt, Germany) and incubated at 35 and 28°C for 24 hr, respectively. If presumptive positive colonies were detected, isolates were confirmed by API 10S (bioMérieux, La Balme les Grottes, France). The method we used in this study was described by Nowak, Mueffling, Caspari, and Hartung (2006).

### 2.9 Enumeration of *Bacillus cereus*

Twenty‐five gram of kimchi was transferred to a sterile stomacher bag containing 225 ml of 0.2% PW, homogenized for 2 min in a stomacher. After homogenization, 1 ml aliquots of homogenized samples were tenfold serially diluted in 9 ml of sterile 0.2% PW, and 0.1 ml of sample or diluent was plated onto a Mannitol Egg Yolk Polymyxin Agar plate (MYP; Difco) and incubated at 30°C for 24 hr. The regulations of the Ministry of Food and Drug Safety of Korea (MFDS) stipulate a contamination level of <3.0 log CFU/g. In the case of *B. cereus*, we simply confirmed whether presumptive colonies were greater or <3.0 log CFU/g.

### 2.10 Kimchi processing analysis: food samples

To assess microbial safety of kimchi processing, we analyzed the kimchi production lines of three different kimchi factories. We also collected samples during each step from raw materials to final product (raw napa cabbage, salted napa cabbage, washed salted napa cabbage, green onions, Asian chives, red pepper flakes, garlic, onions, ginger, glutinous rice flour, salted anchovy sauce, salted shrimp sauce, stock, Korean radishes, washed Korean radishes, shredded washed Korean radishes, and seasoning). Aerobic plate counts and coliforms were enumerated as described previously. In this study, we did not analyze microbial safety of water because all kimchi factories used tap water during processing. Tap water regulations stipulate nondetection of fecal and regular coliforms and *E. coli* in 100 ml and a total aerobic plate count of <2.0 log CFU/ml (Cho & Park, 2012).

### 2.11 Kimchi processing analysis: food contact surfaces

To assess microbial safety of food contact surfaces in kimchi processing, we swabbed food contact surfaces during processing (salting container, dehydration board, conveyor belt, knife, radish cutting machine, and seasoning mixer) with 3M pipette swabs plus (3M Korea Ltd., Seoul, Korea). For enumeration of APC and coliforms, sampled pipette swabs were homogenized for 1 min with a vortexer. After homogenization, 1 ml aliquots of homogenized samples were tenfold serially diluted in 9 ml of sterile 0.2% PW, and 1 ml of sample or diluent was enumerated for APC or coliforms as described previously.

### 2.12 Statistical analysis

All data were repeated three times and analyzed with one‐way ANOVA using the Statistical Analysis System (SAS Institute, Cary, NC, USA) and Duncan's multiple range test to determine whether there were significant differences (*p *<* *0.05) in mean values of microorganism populations. Microbial counts were transformed to log values for analysis. Kimchi processing analysis was conducted only once.

## 3 RESULTS AND DISCUSSION

Table 1 shows salt content, pH, and a_w_ of kimchi samples. Reduced salt content did not affect pH or a_w_ of kimchi. Kimchi normally has salt content <2%, and this may be the main reason for no difference in a_w_. The differences in salt content between regular and reduced‐sodium samples were ca. 1%–2% (Table 1). However, samples from manufacturer C and D showed pH differences between regular and reduced‐sodium kimchi but these differences were statistically insignificant (manufacturer C) or varied according to manufacturing date (manufacturer D).

**Table 1:** Salt content, pH, and a_w_ of kimchi samples

Manufacturers	Product types	Salt content (%)	pH	a_w_
A	Regular	1.9	4.06 ± 0.03^a^ A	0.9901 ± 0.0089^a^ A
	Reduced‐Sodium	1.5	4.03 ± 0.01 A	0.9925 ± 0.0051 A
B	Regular	1.8	3.86 ± 0.01 A	0.9901 ± 0.0005 A
	Reduced‐Sodium	1.3	3.88 ± 0.01 A	0.9884 ± 0.0026 A
C	Regular	2.0	4.39 ± 0.01 A	0.9756 ± 0.0004 A
	Reduced‐Sodium	1.5	4.17 ± 0.04 B	0.9758 ± 0.0027 A
D	Regular	1.8	4.28 ± 0.01 A	0.9865 ± 0.0011 A
	Reduced‐Sodium	1.4	4.99 ± 0.05 B	0.9832 ± 0.0017 A
E	Regular	1.4	4.29 ± 0.04 A	0.9944 ± 0.0053 A
	Reduced‐Sodium	1.2	4.35 ± 0.04 A	0.9932 ± 0.0021 A

a Mean value ± standard deviation. Means with the same letter in the same column per manufacturer and product type are not significantly different (*p *>* *0.05).

Table 2 shows microbial qualities of regular and reduced‐sodium kimchi. There were no significant (*p *>* *0.05) differences in aerobic plate and coliforms counts between regular and reduced‐sodium kimchi. Also, major foodborne pathogens, including *E. coli* O157:H7, *Salmonella* spp., *L. monocytogenes*,* S. aureus*, and *Y. enterocolitica*, were not detected in any sample. In the case of *B. cereus*, regulations of the MFDS of Korea stipulate a contamination level of <3.0 log CFU/g, which is what we observed for all samples. From the data shown in Tables 1 and 2, we confirmed that reducing sodium content did not affect microbial quality of kimchi. However, high levels of coliform contamination were detected in all kimchi samples. Populations of coliforms ranged from 2.11 to 5.15 log CFU/g. Coliforms include pathogenic as well as nonpathogenic bacteria which are readily isolated from common environments such as soil, water, foods, and environmental surfaces. Coliforms are generally used as an indicator of fecal contamination through cross contamination or insufficient food processing (Lee et al., 2009). But, unfortunately, there is no thermal pasteurization step during kimchi processing. Thus, control methods for reducing coliforms in kimchi need to be identified.

**Table 2:** Microbial quality of commercially processed kimchi

Manufacturers	Product types	Microorganism^a^ (log CFU/g)							
		Total aerobic plate counts	Coliforms	*Escherichia coli* O157:H7	*Salmonella* spp.	*Listeria monocytogenes*	*Staphylococcus aureus*	*Yersinia enterocolitica*	*Bacillus cereus*
A	Regular	7.37 ± 0.18 A	3.51 ± 0.33 A	ND^b^	ND	ND	ND	ND	<3.00
	Reduced‐Sodium	7.46 ± 0.15 A	3.56 ± 0.33 A	ND	ND	ND	ND	ND	<3.00
B	Regular	7.81 ± 0.35 A	4.95 ± 0.53 A	ND	ND	ND	ND	ND	<3.00
	Reduced‐Sodium	8.09 ± 0.15 A	5.15 ± 0.81 A	ND	ND	ND	ND	ND	<3.00
C	Regular	8.16 ± 0.11 A	3.16 ± 0.28 A	ND	ND	ND	ND	ND	<3.00
	Reduced‐Sodium	8.19 ± 0.01 A	3.11 ± 0.54 A	ND	ND	ND	ND	ND	<3.00
D	Regular	7.83 ± 0.13 A	4.45 ± 0.13 A	ND	ND	ND	ND	ND	<3.00
	Reduced‐Sodium	8.00 ± 0.08 A	4.36 ± 0.38 A	ND	ND	ND	ND	ND	<3.00
E	Regular	5.76 ± 0.07 A	2.11 ± 0.19 A	ND	ND	ND	ND	ND	<3.00
	Reduced‐Sodium	5.73 ± 0.08 A	2.49 ± 0.44 A	ND	ND	ND	ND	ND	<3.00

a Mean value ± standard deviation. Means with the same letter in the same column per manufacturer and product type are not significantly different (*p *>* *0.05).

b ND not detected, <1/25 g (qualitative enrichment).

To reduce the level of coliforms contamination, we analyzed specific kimchi producing processes to find microbial hazard factors. The results are shown in Tables 3, 4, 5, 6. Manufacturers D and E refused to disclose their processing steps. Using this analysis, we were able to identify five control points during kimchi processing. Using antimicrobial agents for washing napa cabbage is a first control point for reducing coliform levels of kimchi samples. Tables 3 and 4 show the microbial qualities of samples which were collected at each kimchi processing step. The APC and coliforms counts of raw napa cabbage were 5.90–6.90 log CFU/g and 1.60–2.48 log CFU/g, respectively. Even though raw napa cabbage showed high levels of microbial contamination, most manufacturers did not wash raw cabbage prior to kimchi production or use antimicrobial agents during the washing process. Fukuyama et al. (2009) reported that 100 ppm sodium hypochlorite (NaClO) for 10 min reduced *E. coli* O157:H7 by 2.69 log CFU/g on shredded cabbage. And Inatsu et al. (2017) reported that washing with 100 ppm NaClO for 5 min resulted in the reduction of APC and coliforms on shredded cabbage by 1.8 and 1.6 log CFU/g, respectively. A processing step using antimicrobial agents for washing raw napa cabbage is needed to reduce the microbial load.

**Table 3:** Total aerobic plate counts (log CFU/g) of food samples collected during kimchi processing

Samples	Manufacturers		
	A	B	C
Raw napa cabbage	6.00	5.90	6.90
Salted napa cabbage	4.00	6.83	6.00
Washed salted napa cabbage	3.60	6.25	5.43
Green onions	7.26	7.01	ND
Asian chives (buchu)	6.15	NI	NI
Red pepper flakes	6.63	6.20	6.30
Garlic	6.28	7.49	ND
Onions	5.43	6.80	ND
Ginger	4.15	8.66	ND
Glutinous rice flour	<1.00	7.29	4.78
Salted anchovy sauce	3.00	4.73	<1.00
Salted shrimp sauce	<1.00	NI	NI
Stock (kelp or dried pollack)	5.83	8.10	3.00
Korean radishes	5.00	5.20	6.08
Washed Korean radishes	4.60	5.58	6.18
Shredded washed Korean radishes	5.26	7.48	5.11
Seasoning	5.60	6.76	6.77

Note

ND: not determined; NI: not included in kimchi formulation.

**Table 4:** Coliforms (log CFU/g) of food samples collected during kimchi processing

Samples	Manufacturers		
	A	B	C
Raw napa cabbage	1.60	2.48	2.15
Salted napa cabbage	2.04	3.26	2.00
Washed salted napa cabbage	2.04	2.64	2.64
Green onions	6.15	4.54	ND
Asian chives (buchu)	4.28	NI	NI
Red pepper flakes	3.00	4.18	2.70
Garlic	<1.00	3.08	ND
Onions	1.60	5.54	ND
Ginger	2.80	5.48	ND
Glutinous rice flour	<1.00	4.11	2.00
Salted anchovy sauce	<1.00	4.41	<1.00
Salted shrimp sauce	<1.00	NI	NI
Stock (kelp or dried pollack)	2.90	4.53	<1.00
Korean radishes	2.15	2.26	5.79
Washed Korean radishes	2.04	5.20	5.95
Shredded washed Korean radishes	2.64	5.18	4.28
Seasoning	2.85	4.36	3.76

Note

ND: not determined; NI: not included in kimchi formulation.

**Table 5:** Total aerobic plate counts (log CFU/cm^2^) of food contact surfaces swabbed during kimchi processing

Food contact surfaces	Manufacturers		
	A	B	C^a^
Salting container	3.00	3.60	ND
Dehydration board	6.48	6.87	ND
Conveyor belt	5.11	5.57	ND
Knife	5.68	5.43	ND
Radish cutting machine	6.52	6.23	ND
Seasoning mixer	5.48	4.87	ND

a Manufacturer C declined analysis of food contact surfaces.

**Table 6:** Coliforms (log CFU/cm^2^) of food contact surfaces swabbed during kimchi processing

Food contact surfaces	Manufacturers		
	A	B	C^a^
Salting container	<1.00	1.85	ND
Dehydration board	<1.00	4.46	ND
Conveyor belt	4.37	2.60	ND
Knife	2.18	2.41	ND
Radish cutting machine	3.15	2.38	ND
Seasoning mixer	2.40	2.48	ND

a Manufacturer C declined analysis of food contact surfaces.

A second control point for reducing the microbial contamination load of kimchi is washing salted napa cabbage with appropriate sanitizers, such as organic acids, electrolyzed water, or sodium hypochlorite. APC counts of salted napa cabbage and washed salted napa cabbage were 4.00 to 6.83 and 3.60 to 6.25 log CFU/g, and coliforms counts of those samples were 2.00 to 3.26 log CFU/g and 2.04 to 2.64 log CFU/g, respectively. Microbial contamination levels were not appreciably reduced after the washing step because tap water without sanitizers was used for washing. The main purpose of the washing step is to remove excess salt content of salted napa cabbage. However, Park, Kim, and Oh (2016) reported that washing salted napa cabbage with 30 ppm slightly acidic electrolyzed water reduced the total microbial count in salted Chinese cabbage by about 2.25 log CFU/g. Application of sanitizer during salting is also recommended. Kim et al. (2015) used phytic acid to reduce *E. coli* O157:H7 and coliforms in napa cabbage destined for kimchi producing. In the hyper salting step, adding 2% phytic acid resulted in a five log reduction of *E. coli* O157:H7, and coliforms were reduced to under the detection limit (1.0 log CFU/g).

The third control point consists of control methods for ensuring microbial safety of kimchi seasoning. Various submaterials (green onions, Asian chives, red pepper flakes, garlic, onions, ginger, glutinous rice flour, salted anchovy sauce, salted shrimp sauce, stock of kelp or dried pollack, and shredded Korean radish) are used to season kimchi. Total aerobic plate and coliforms counts of each supplemental ingredient can affect the microbial population of the finished kimchi product. The microbial contamination levels of submaterials used by manufacturer A were less than those of manufacturers B and C. The coliforms counts of seasoning and final product of manufacturer A (green onions, Asian chives, red pepper flakes, garlic, ginger, glutinous rice flour, salted anchovy sauce, salted shrimp sauce, kelp stock, and Korean radishes) were 2.86 and 3.55 log CFU/g, respectively, whereas those of manufacturer B (green onions, red pepper flakes, garlic, ginger, glutinous rice flour, salted anchovy sauce, kelp stock, and Korean radishes) were 4.36 and 5.15 log CFU/g, respectively, and manufacturer C (green onions, red pepper flakes, garlic, ginger, glutinous rice flour, salted anchovy sauce, salted shrimp sauce, kelp stock, Korean radishes) showed 4.00 and 3.76 log CFU/g, respectively. To ensure microbial safety of kimchi seasoning, several methods were developed. Park et al. (2016) reported that washing Korean radishes and green onions with slightly acidic electrolyzed water (30 ppm) reduced total bacterial counts by about 1.52 and 0.92 log CFU/g, respectively. Na and Park (2003) reported that horseradish powder reduced *E. coli* in kimchi seasoning by 1.0 log CFU/g. And fermentation of kimchi seasoning helps to reduce the coliforms. Song, Cheon, Yoo, Chung, and Seo (2016) also reported that storage of kimchi seasoning at 4°C for 2 weeks reduced coliforms to nondetectable levels.

To prevent cross contamination of kimchi, through sanitization of food contact surfaces could be a fourth control point to ensure microbial safety of kimchi. Tables 5 and 6 show the microbial counts of food contact surfaces which were sampled during each kimchi processing step. Manufacturer C declined to analyze the food contact surfaces. Most investigated food contact surfaces showed more than 5 log CFU/cm^2^ of APC and over 2 log CFU/cm^2^ of coliforms. Sheen and Hwang (2010) reported that ready‐to‐eat meat products can be cross‐contaminated with *E. coli* O157:H7 while slicing with contaminated blades. Jensen, Friedrich, Harris, Danyluk, and Schaffner (2013) also reported that pathogens (*Salmonella* and *E. coli* O157:H7) were transferred from food contact surfaces (ceramic, glass, plastic, stainless steel) to carrots, celery, lettuce, and watermelons.

The last (fifth) control point for reducing coliforms in kimchi is ripening (fermentation). There are several studies which confirm that coliforms can be decreased during the ripening step. Choi, Hwang, Hong, and Lee (2016) reported that coliforms were reduced to nondetectable levels after 2 weeks of fermentation at 4°C. Kwon and Kim (2007) also reported that coliforms in kimchi samples decreased to nondetectable levels after 9–12 days of ripening at 4°C. Conversely, some studies showed that no changes or even increases in coliforms levels occurred during the ripening period. Kim and Yoon (2005) reported that coliforms increased during ripening of kimchi at 10°C. Cheon et al. (2015) reported that coliforms in kimchi increased after 2 weeks of storage at 4°C. Knarreborg, Miquel, Granli, and Jensen (2002) reported that lactic acid‐induced growth of coliforms at pH 5.5, but at pH 4.5, it could reduce the population of coliforms. Taken together, a fast pH drop at the early phase of kimchi ripening is one of the key factors for reducing coliforms. Choi, Lee, Kim, Lee, Kim, Lee, Ha, Oh, Choi, et al. (2018) and Choi, Lee, Kim, Lee, Kim, Lee, Ha, Oh, Yoon, et al. (2018) reported that fermentation of kimchi at a higher temperature resulted in a fast pH drop. In these studies, the faster the pH decrease of kimchi, the faster the reduction of pathogenic *E. coli* and *Salmonella*. But the exact effect of fast ripening on microbial populations should be examined in future studies.

In conclusion, this study evaluated the microbial safety of reduced‐sodium Korean kimchi products. Reduced‐sodium kimchi did not show a higher microbial load than that of regular kimchi. However, high levels of coliforms contamination were observed in both kinds of kimchi samples. Through analyzing specific factors in the production line, we found that five key points for reducing coliforms levels during kimchi processing are needed: (a) washing raw napa cabbage with sanitizer, (b) washing salted napa cabbage with sanitizer, (c) washing submaterials with sanitizer for reducing coliforms in kimchi seasoning, (d) maintaining clean food contact surfaces, and (e) fast ripening. The results of this study could be helpful for the kimchi industry to produce safe reduced‐sodium kimchi products. However, further investigations need to be performed to determine the proper sanitizers for each step and the effect of various ripening methods on the microbial quality of kimchi.

## CONFLICT OF INTEREST

The authors declare that they have no competing interest.

## ETHICAL STATEMENTS

This study does not involve any human or animal testing.
